# Pixelated Vacuum Flat Panel Detector Using ZnS Photoconductor and ZnO Nanowires Cold Cathode

**DOI:** 10.3390/nano12050884

**Published:** 2022-03-07

**Authors:** Delin Hu, Xingpeng Bai, Chengyun Wang, Zhipeng Zhang, Xiaojie Li, Guofu Zhang, Shaozhi Deng, Jun Chen

**Affiliations:** State Key Laboratory of Optoelectronic Materials and Technologies, Guangdong Province Key Laboratory of Display Material and Technology, School of Electronics and Information Technology, Sun Yat-sen University, Guangzhou 510275, China; hudlin@mail2.sysu.edu.cn (D.H.); baixp3@mail2.sysu.edu.cn (X.B.); wangchy75@mail2.sysu.edu.cn (C.W.); zhangzhp25@mail.sysu.edu.cn (Z.Z.); lixj67@mail2.sysu.edu.cn (X.L.); zhanggfu@mail.sysu.edu.cn (G.Z.); stsdsz@mail.sysu.edu.cn (S.D.)

**Keywords:** vacuum flat panel detector, ZnS photoconductor, ZnO nanowires, cold cathode

## Abstract

Vacuum flat panel detectors (VFPDs) using cold cathode have important applications in large-area photoelectric detection. Based on the electron-bombardment-induced photoconductivity (EBIPC) mechanism, the photoconductor-type VFPDs achieved high detection sensitivity. However, pixelated imaging devices have not yet been developed. In this paper, we fabricate a 4 × 7 pixel vacuum flat panel detector array made of ZnS photoconductor and ZnO nanowires cold cathode for an imaging application. The responsivity of the device and the pixel current uniformity are studied, and imaging of the patterned objects is achieved. Our results verify the feasibility of VFPDs for imaging.

## 1. Introduction

X-ray imaging is widely used in industrial inspection [1], security screening [2], medical diagnosis, [3,4] etc. The large-area flat panel photodetector is the key component of X-ray imaging. The demand for low-dose medical X-ray imaging [5] has inspired extensive studies on high-sensitivity flat panel X-ray detectors [6,7].

X-ray detectors can be divided into two categories, i.e., direct and indirect conversion X-ray detectors. The direct conversion X-ray detector uses high-efficiency photoconductive materials to generate electron-hole pairs (EHPs) directly from X-ray irradiation. The direct conversion X-ray detectors rely on high-efficiency X-ray photoelectric conversion material, such as a-Se, perovskite, CdTe [8,9,10,11,12], etc. [13,14,15]. Silicon-based X-ray detectors, such as lithium drifted silicon detector (Si (Li)) [16] and silicon drift detector (SDD) [17] are also used, especially in X-ray spectroscopy. For the indirect conversion X-ray detectors, the incident X-rays are first converted into visible light by the scintillator, and then the visible light is converted into EHPs by the photoconductor or photodiode [18,19]. Because of lacking proper materials, the commercial use of large-area direct conversion X-ray detectors is still limited. Nowadays, the majority of commercial large-area X-ray detectors are based on indirect conversion detectors, which use a photoconductor or photodiode integrated with thin-film transistor (TFT) arrays [20], or a complementary metal-oxide-semiconductor (CMOS) to read out the photoelectric signal and realize imaging [21]. To optimize the sensitivity, some studies were dedicated to looking for new pixel architecture like active pixels which integrate additional in-pixel amplifier TFT [22], or new multiplication mechanisms [23], such as the avalanche mechanism [24], in which impact ionization under a high electric field is used to increase detection efficiency. 

In recent years, vacuum devices are also studied for photodetection due to the advantages of high-sensitivity and high resistance to radiation [25]. The main structure of vacuum photodetectors includes a photoconductor anode and a cold cathode field emitter array (FEA), which can be made into a large-area flat panel detector. The concept of a flat panel detector based on field emitter arrays can be dated back to the work of Kurashige et al. in the early 1990s [9]. In 1997, Yamagishi et al. developed a vacuum flat panel detector for visible light imaging, which is made up of FEAs and high gain avalanche rushing amorphous photoconductor (HARP) [26]. The NHK team from Japan extensively studied HARP vacuum flat panel detectors using Spindt-type FEA for high-sensitivity and high-resolution TV camera tube applications in 2002 [27]. Two years later, Zhao et al. reported that they realized indirect conversion vacuum flat panel X-ray imaging by using a-Se as a photoconductor [28]. Recently, Zhang et al. reported vacuum flat panel detectors using ZnS photoconductor based on the electron-bombardment-induced photoconductivity (EBIPC) mechanism for possible high-sensitivity X-ray detection application [29]. The VFPD using ZnS photoconductor and ZnO nanowire field emitters achieved a high gain up to 10^4^ [29,30]. The EBIPC mechanism indicates that light excitation acts as a trigger and generates initial EHPs, and electron beam bombardment causes an iterative multiplication of carriers. Recently, they also reported a sensitive direct conversion X-ray detector formed by ZnO nanowire field emitters and β-Ga_2_O_3_ photoconductor target with the EBIPC mechanism, which can achieve a high internal gain (2.9 × 10^2^) and high detection sensitivity (3.0 × 10^3^ μCGy^−1^_air_ cm^−2^) for a 6 keV X-ray at the electric field of 22.5 V μm^−1^ [31,32]. Despite the devices based on the EBIPC mechanism exhibiting excellent performance in high-sensitivity detection, the imaging devices have not been reported yet.

In this work, we fabricate photoconductor-type vacuum flat panel detectors with 4×7 pixel arrays using ZnS photoconductor and ZnO nanowire field emitters. As aimed at an indirect conversion X-ray detection, the performance of the VFPD is characterized by visible light irradiation without the scintillator. The photocurrent of the pixels under different light intensities and voltages is measured, from which we calculate the current gain, responsivity and uniformity of the pixels. Furthermore, we use the detector to eventually achieve imaging of the objects. 

## 2. Experimental

**Figure 1:**
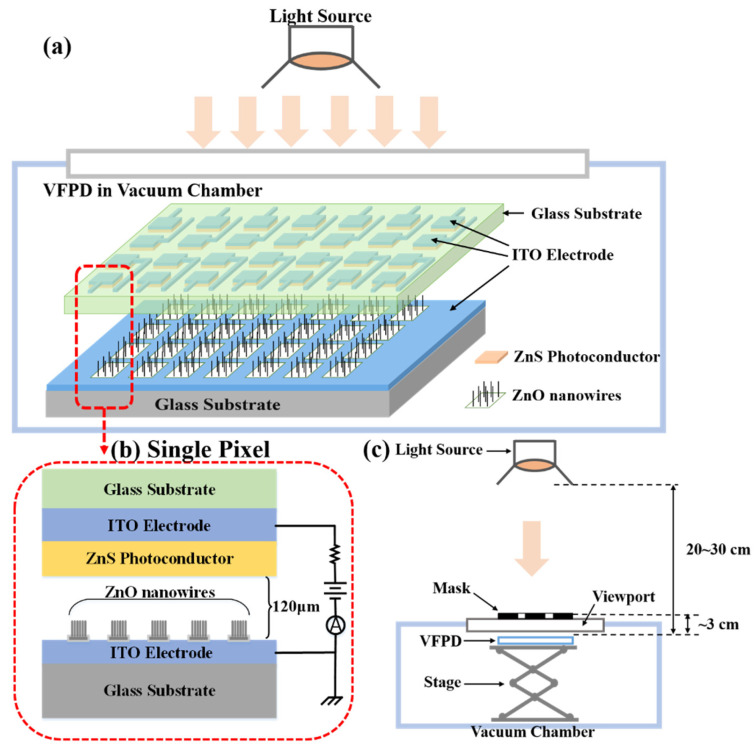
(**a**) Illustration of the structure and photoresponse measurement set-up for VFPD; (**b**) Structure of a single pixel; (**c**) Set-up for imaging measurement of the vacuum flat panel detector.

Figure 1a,b shows the schematic illustration of vacuum flat panel detectors and the structure of a single pixel. The fabricated detector is composed of an anode panel and a cathode panel. Furthermore, 4 × 7 pixel arrays with a single pixel size of 6 mm × 8 mm are formed by patterning the anode with pixelated ZnS photoconductor on an indium tin oxide (ITO)-coated glass substrate. The detector pixels are fabricated on an area of 4.5 cm × 8.0 cm. The cathode is composed of patterned ZnO nanowire FEAs on an ITO-coated glass substrate. The anode pixel is individually connected to the readout circuits by the ITO electrode and can be biased with voltage, while all the cathode FEAs are connected to the ground. Each single pixel is a vacuum diode-type unit that can work independently. A 120 μm vacuum gap is formed between the anode and cathode panels by ceramic spacers.

The fabrication process of the VFPD using ZnS photoconductor and ZnO nanowire field emitters is similar to our early studies [29,30]. For the anode, the ITO film is deposited on the glass substrate by DC magnetron sputtering, and the ZnS photoconductor is deposited on the ITO-coated glass by electron beam evaporation with a deposition rate of 4 nm/s. For the cathode, a Zn film is first deposited on an ITO-coated glass substrate using electron beam evaporation and patterned using photolithography and the lift-off process. The ZnO nanowires are grown using thermal oxidation under 470 °C for 3 h. The morphologies of the ZnS photoconductor and ZnO nanowires are observed using a scanning electron microscope (SEM; Zeiss SUPRA 60, Oberkochen, Germany). 

The photoresponse of the fabricated detectors is measured in a vacuum chamber with a pressure of ~1.0 × 10^−5^ Pa. A metal halide lamp (Philips, CDM-T 70W, 400~1100 nm, Foshan, China) is used as the light source to illuminate the device for measuring the photocurrent. The performance of the detector is characterized by visible light irradiation without the scintillator. The light source is placed above the device, as shown in Figure 1a. The optical power is adjusted and measured by an optical power meter (Thorlabs, PM100D, S120VC, New Jersey, CA, USA). The voltage between the anode and cathode is adjusted from 0 to 2200 V using a high voltage source meter (Keithley, 2657A, OH, USA). The photocurrent and the dark current are also recorded using the source meter. The current of 28 pixels is measured separately. For imaging measurement, designed patterns are projected onto the detector through a mask, as shown in Figure 1c. The light source is placed about 20~30 cm above the device. A metal letter mask is attached to the glass viewport of the vacuum chamber. The device is placed below the viewport at a distance of ~3 cm. The current of each pixel is measured separately with the source meter, and imaging is reproduced by using a home-designed imaging program. 

## 3. Results & Discussion

**Figure 2:**
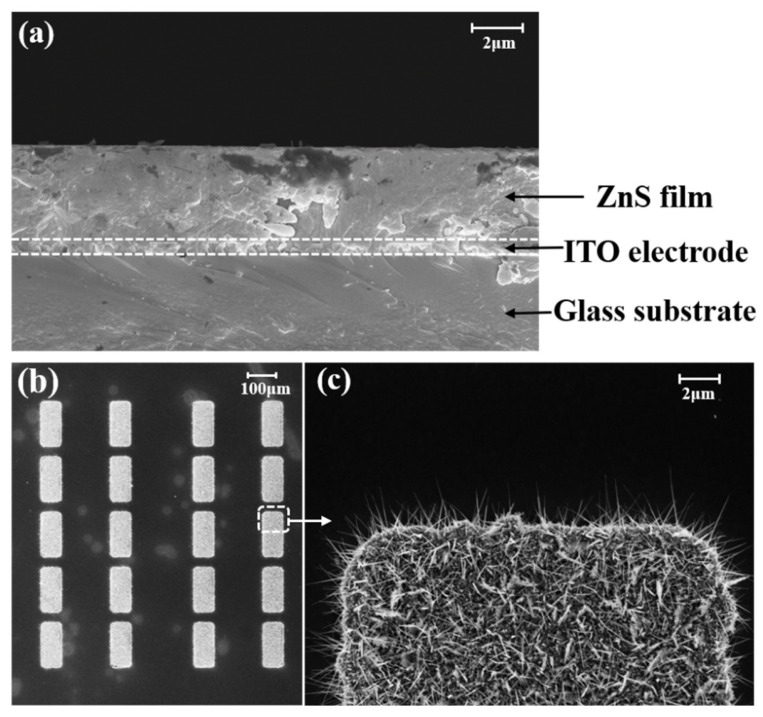
SEM images. (**a**) Cross-sectional view of ZnS photoconductor anode. The dotted line indicates the layer of ITO electrode; (**b**) Top-view of patterned ZnO nanowire FEAs; (**c**) Showing the morphology of the ZnO nanowires in the selected area of (**b**).

Figure 2a shows the cross-sectional morphology of the anode panel. The thickness of the glass substrate for the anode and cathode is 3 mm. The ZnS film was prepared on a 500 nm thick ITO electrode on the glass substrate. The ZnS film has a thickness of 3.3 μm, which is an optimized thickness for detection using the EBIPC mechanism [30]. Figure 2b,c shows the morphology of the ZnO nanowire cathode under different magnifications. The cathode is composed of ZnO nanowire FEAs and there are nearly 2000 patterned ZnO nanowires in a single pixel with each size of 25 μm × 60 μm. The nanowire has an average length of 3~4 μm and a tip diameter of ~20 nm. The population density of the nanowire is about ~5 × 10^8^ cm^−2^.

**Figure 3:**
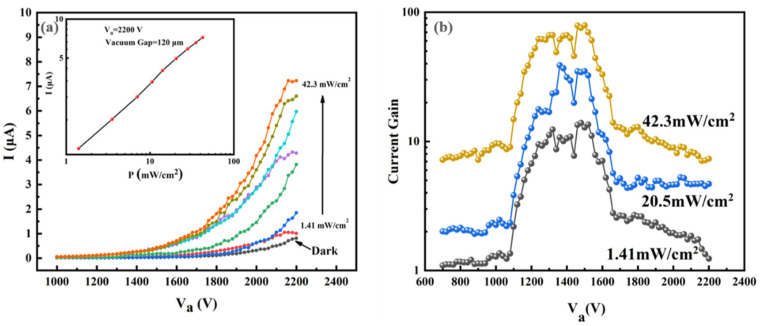
(**a**) Photoresponse of a single pixel of the vacuum flat panel detector (I-V_a_ plot in the dark and under different light intensities); inset shows I-P plot @ V_a_ = 2200 V; (**b**) the current gain versus anode voltage under different light intensities.

The emission current of one pixel as a function of applied anode voltage is measured in the dark and under the illumination of different light intensities. The light power densities range from 1.41 to 42.3 mW/cm^2^. Figure 3a shows the typical current-anode voltage (I-V_a_) curves obtained from one pixel. The current versus optical power density is shown in the inset of Figure 3a for an anode voltage of 2200 V. The current gradually increases as the light power densities increase, which is nearly 7.5 μA at 42.3 mW/cm^2^, while the dark current is less than 1.0 μA when the anode voltage is 2200 V. Within the light power density range of 1.41~42.3 mW/cm^2^, the detector exhibits a good linear photoresponse.

We further calculate the current gain of the pixel when the anode voltage changes from 700 to 2200 V under different light intensities. The current gain is calculated using $\Delta I/I_{dark}$. The ΔI was defined as $I_{light}-I_{dark}$, in which $I_{light}$ is the photocurrent and $I_{dark}$ is the dark current. Figure 3b shows the curves of current gain versus bias voltage under light intensities of 1.41 mW/cm^2^, 20.5 mW/cm^2^ and 42.3 mW/cm^2^, showing that the higher the light intensity, the greater the current gain. In addition, the current gain increases initially and then decreases under a certain light intensity. This phenomenon can be explained by the EBIPC mechanism. According to the mechanism, the collection efficiency *η* of the photo-generated carriers can be estimated using the following equation [12]:

(1) $$\eta =\frac{\mu_{n}\tau E}{L}\left(1-e^{-\frac{L}{\mu_{n}\tau E}}\right),$$

where $\mu_{n}$ is the electron mobility, τ is the lifetime of the carriers, *E* is the electric field across the photoconductor and *L* is the thickness of the photoconductor which was 3.3 μm in this study.

The collected carriers $\Delta n_{L}^{n}$ due to the light incidence and following-up EBIPC mechanism can be expressed as:

(2) $$\Delta n_{L}^{n}=\left(\Delta n_{ph}+\Delta n_{e}\right)\;\eta ,$$

where $\Delta n_{ph}$ is induced by the light excitation and $\Delta n_{e}$ relates to the electron beam bombardment induced carriers.

When the anode voltage increases, the electron-multiplying effect is enhanced and a rise in the current gain will be observed. The charge collection efficiency η initially increases rapidly and eventually reaches a maximum value, so that the photocurrent caused by $\;\Delta n_{L}^{n}$ increases rapidly first and then increases slowly. In the meantime, the dark current increases gradually as the voltage increases. The saturation of the charge collection efficiency η and the increase in the dark current eventually causes the current gain to decrease. Therefore, a maximum value of current gain will occur. In this work, the maximum current gain reaches 83.9 under a light power density of 42.3 mW/cm^2^, which is comparable to the previous work [30].

**Figure 4:**
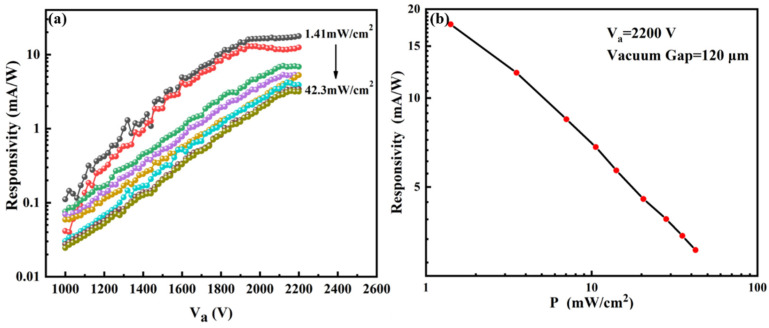
The responsivity of single pixel: (**a**) the responsivity versus V_a_ plot; (**b**) the responsivity versus power density when V_a_ = 2200 V.

The responsivity R is calculated using R=ΔI/(P·S) with the unit of mA/W. P is the light power density, and its unit is mW/cm^2^. S is the light-receiving area of the pixel unit with the size of 6.0 × 8.0 mm^2^. Figure 4a shows the responsivity of a single pixel under different light intensities and anode voltages. The maximum responsivity at 1.41 mW/cm^2^ is about 18 mA/W, while the responsivity at 42.3 mW/cm^2^ is 3.2 mA/W. In Table 1, we summarize the performance of some reported photodetectors benchmarked by ultraviolet-visible light. Compared with a previously reported VFPD using ZnS photoconductor [30], the responsivity is low because a small vacuum gap is used in the present study, which increases the dark current.

Moreover, it is found that the responsivity increases with the anode voltage and gradually ceases to increase as the voltage increases to nearly 2100 V, which was more pronounced in lower light intensity. This phenomenon can be explained by the EBIPC mechanism. As the electric field increases, the collection efficiency of carriers will reach a maximum value while the photo-generated carriers under a certain light intensity are limited. Therefore, the responsivity of the pixels will reach a constant value instead of increasing continuously. The smaller the light intensity, the smaller the applied electric field required to establish this equilibrium state, which can explain why this phenomenon is more pronounced when the light intensity is lower. 

Figure 4b shows the variation in responsivity of a single pixel with light power density under an anode voltage of 2200 V. The responsivity decreases as light intensity increased, and a linear relation is observed. 

**Figure 5:**
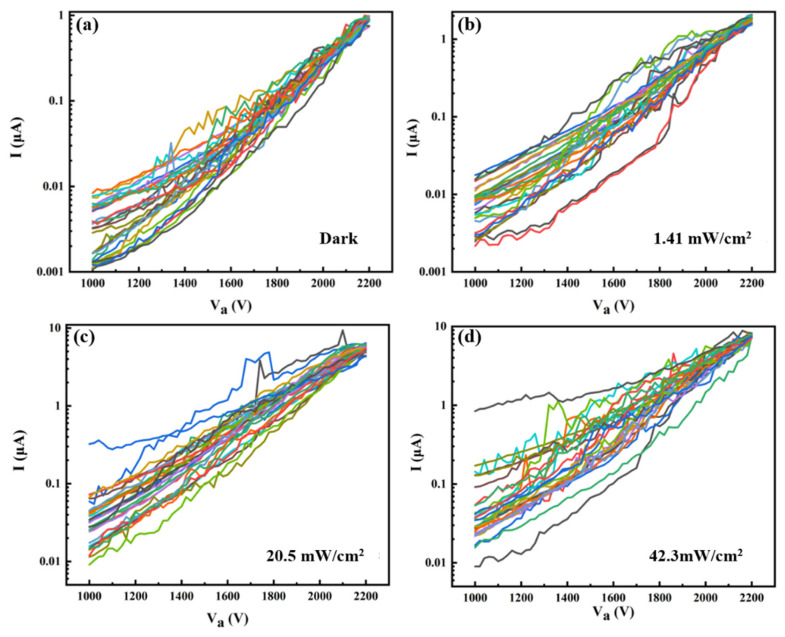
The I-V_a_ curves of 28 pixels under different light intensities. (**a**) in the dark; (**b**) 1.41 mW/cm^2^; (**c**) 20.5 mW/cm^2^; (**d**) 42.3 mW/cm^2^.

**Figure 6:**
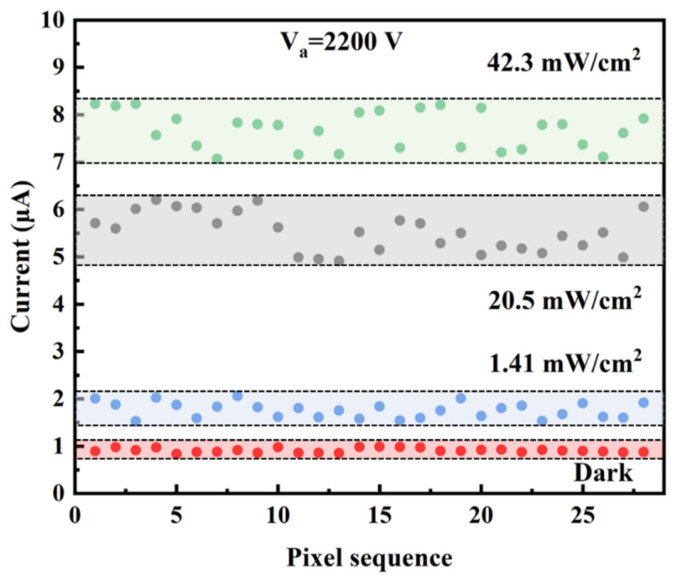
Scattered distribution diagram of current for 28 pixels under different light intensities when the anode voltage is 2200 V.

To verify the feasibility of the detector for imaging application, the uniformity of the device is studied. The I-V_a_ curves of 28 pixels under different light intensities are shown in Figure 5, which indicates the same changing trend. The relative difference of each pixel current decreases as the voltage increases. The better uniformity observed at higher voltage relates to the self-ballasting effect in the field emission from ZnO nanowires. The ZnO nanowires are semiconductors and have resistance. When the voltage increases, the emission current increases and the voltage on the ZnO nanowire will increase. Those nanowires with a higher emission current will have a higher voltage fall, which will, in turn, lower the vacuum voltage applied to the tip of the nanowire and suppress the emission. This will lead to a more uniform emission. To compare the current uniformity of each pixel more directly, the pixel currents at the anode voltage of 2200 V are chosen to make a scattered distribution diagram, as shown in Figure 6. The horizontal axis is the serial number of the pixel and the band-shaped area is the distribution interval of the pixel current. It is clear that the range of the band-shaped area under higher light intensity is larger than that under weak light. The mean average current and the variance were calculated, which are 0.910 (±0.002) μA, 1.76 (±0.03) μA, 5.52 (±0.22) μA, 7.68 (±0.15) μA for the light intensity of dark, 1.41, 20.5 and 42.3 mW/cm^2^, respectively. In addition, we calculate the average responsivity under light power densities of 1.41 mW/cm^2^, 20.5 mW/cm^2^ and 42.3 mW/cm^2^ at an anode voltage of 2200 V. The obtained average responsivities are 17.2 mA/W, 7.04 mA/W and 2.56 mA/W, respectively.

**Figure 7:**
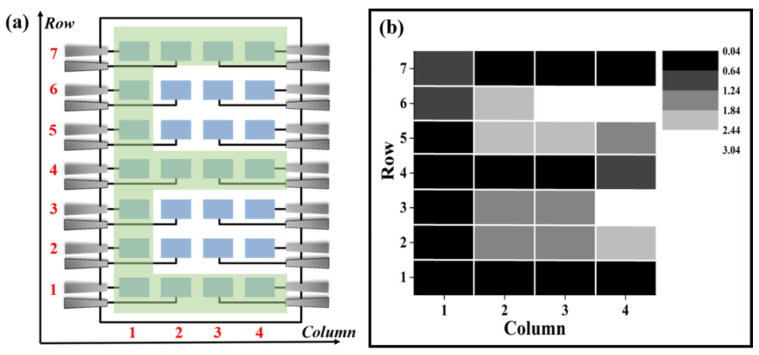
(**a**) Schematic diagram of pixel arrangement for imaging; (**b**) Obtained grayscale image of letter “E” under light power density of 20 mW/cm^2^.

The good overall uniformity of pixels provides the possibility for the following grayscale imaging experiment, in which the operating voltage of the detector is set to 2200 V and the light power density was set to 20 mW/cm^2^. We choose 2200 V as the voltage for imaging because the responsivity at 2200 V reaches a maximum value, as shown in Figure 4a. Moreso, the current uniformity of the pixel is better. Figure 7a shows the schematic for the imaging experiment using the 4 × 7 pixel array. A pattern of an English letter of the alphabet is illuminated by the light source and projected onto the device, which forms a shadow on the letter-covered pixels. The current of each pixel is collected individually and images are reconstructed directly using the current value. The grayscale is formed by assuming the highest current is 100% white and the lowest current is 100% dark. Figure 7b shows the results for the imaging results of the letter “E”, which shows the E can be well detected.

**Figure 8:**
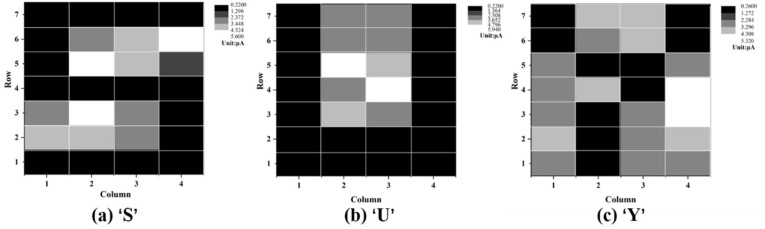
Imaging results of several letter patterns. (**a**) S; (**b**) U; (**c**) Y. The light power density is 40 mW/cm^2^.

To further verify the imaging effect and the stability of the device, we tested several different patterns under the light power density of 40 mW/cm^2^. The results are shown in Figure 8. In these images, the corresponding patterns can be clearly distinguished. Although we can get a relatively clear image of the corresponding pattern, there is still some nonuniformity in the imaging, which we think can be improved by optimizing the preparation process. On the other hand, the nonuniformity of the pixels can be compensated by means of digital signal calibration considering the responsivity of each pixel.

## 4. Conclusions

In this paper, we fabricate pixelated vacuum flat panel detectors using ZnS photoconductor and ZnO nanowires field emitter arrays. The photoresponse of individual pixels and the uniformity of pixels are studied, which shows good stability and consistency. Furthermore, clear imaging of letter patterns is achieved. Our results confirm the application feasibility of vacuum flat panel detectors.

## Figures and Tables

**Table 1:** Comparison of performance of some reported photodetectors.

Photodetector	Area	Responsivity (mA/W)	Light (nm); P (mW/cm^2^)	Imaging Capability	Ref.
VFPD using pixelated ZnS photoconductor	4.5 × 8.0 cm^2^ (6 × 8 mm^2^/pixel)	18	white light; 1.41	Yes	This work
VFPD using ZnS photoconductor	4.5 × 8.0 cm^2^	177	white light; 1	No	[30]
ZnS-MoS_2_ hybrid photodetector	2 × 0.5 cm^2^	1.785 × 10^−2^	white light; 19.1	No	[33]
Se/n-Si heterojunction photodetector	1.41 × 10^−1^ mm^2^	37.4	610; 0.704	No	[34]
Perovskite photodetector	not given	12.7 × 10^3^	white light; 3.2 × 10^−2^	No	[35]
Se/ZnO heterojunction photodetector	~1 × 1 cm^2^	2.65	370; 0.85	No	[36]

## Data Availability

Data are contained within the article.
